# Correlation of nutrition with neuropathy in ovarian cancer patients

**DOI:** 10.6026/973206300212328

**Published:** 2025-08-31

**Authors:** Anirudh Singh, Vikas Asati, Rajesh Patidar, Hemendra Mishra, Chitalkar P.G., Arth Shah, Astha Parmar, Archit Jain

**Affiliations:** 1 Department of Medical Oncology, Sri Aurobindo Institute of Medical Sciences, Indore, Madhya Pradesh, India

**Keywords:** Chemotherapy-induced peripheral neuropathy (CIPN), L3 skeletal muscle area (SMA), L3 skeletal muscle index (SMI), nerve conduction velocity, ovarian cancer, paclitaxel, sarcopenia

## Abstract

The relationship between nutritional parameters (L3 SMI and albumin) and the incidence of chemotherapy-induced peripheral neuropathy (CIPN) in ovarian cancer patients treated with paclitaxel and carboplatin. The study included 53 patients and logistic regression identified age, height, TSH and hemoglobin levels as independent predictors of neuropathy. NCV detected subclinical neuropathy in patients without overt symptoms. We show that NCV is a valuable tool for early detection of CIPN, especially in high-risk patients and highlights the need for larger studies to explore the role of inflammatory markers in this context.

## Background:

Ovarian carcinoma, a common gynecologic cancer, remains a significant cause of morbidity and mortality in women [1]. Paclitaxel and carboplatin are the cornerstone treatments for ovarian cancer, but chemotherapy-induced peripheral neuropathy (CIPN), especially with paclitaxel, presents a major dose-limiting toxicity [2]. Various patient-specific factors, such as malnutrition, sarcopenia and altered body composition, influence the onset and severity of neuropathy [3]. Reduced muscle mass and fat reserves can exacerbate chemotherapy-related toxicity by affecting drug metabolism and pharmacokinetics [4]. Sarcopenia, often quantified by the L3 Skeletal Muscle Index (SMI), is a common complication in cancer patients and can serve as a predictor for chemotherapy-induced adverse effects [5]. Although ovarian cancer is often associated with malnutrition, its link to CIPN has not been consistently established [6]. This study aims to investigate the correlation between malnutrition, assessed through L3 SMI and serum albumin levels and the occurrence of CIPN in ovarian cancer patients receiving paclitaxel and carboplatin. In addition, the study will examine the potential contributions of pre-existing hypothyroidism and vitamin B12 deficiency to the severity of neuropathy. Therefore, it is of interest to describe how nutritional status and underlying conditions, such as hypothyroidism and vitamin B12 deficiency, may influence chemotherapy-related neurotoxicity, offering potential insights for improving patient care.

## Methodology:

This prospective observational study was conducted on 53 patients diagnosed with ovarian carcinoma who were undergoing chemotherapy with paclitaxel and carboplatin. The inclusion criteria required participants to have histologically confirmed ovarian carcinoma and to have received at least six cycles of paclitaxel and carboplatin. Baseline fat thickness assessment, including L3 vertebra imaging, was performed before the start of chemotherapy. Patients with pre-existing neuropathies due to diabetes, alcoholism, or other neurological disorders, as well as those receiving concurrent neurotoxic agents, were excluded from the study. Data collection involved a nutritional assessment, including the measurement of L3 vertebra fat thickness through cross-sectional imaging. Neuropathy was assessed using Nerve Conduction Velocity (NCV) after the completion of six chemotherapy cycles. Additionally, patients were asked to report the presence of neuropathies based on symptoms such as tingling or a sensation of pins and needles. Other measurements included Body Surface Area (BSA), which was calculated using the Mosteller formula. Baseline and post-chemotherapy thyroid profiles (TSH, T3 and T4), serum albumin and vitamin B12 levels were also evaluated. The cumulative doses of paclitaxel and carboplatin were documented over the six cycles. Data analysis aimed to determine the association between the presence of neuropathy and nutritional levels. A Spearman correlation was conducted to assess the relationship between L3 Skeletal Muscle Index (SMI) and neuropathy grade. Given the sample size of 53 patients, neuropathy was classified as either present or absent (1 or 0, respectively) for both NCV and clinical qualitative reports. Logistic regression was performed to predict the effects of independent variables such as age, TSH, L3 SMI and hemoglobin (Hb) on the presence of neuropathy, as measured by NCV.

## Results:

This prospective observational study included 53 patients with histologically confirmed ovarian carcinoma treated with paclitaxel-carboplatin. The female patients were aged from 35-80 years with a mean age of 53.5 years (±10.5, median 53). The height ranged from 135-159cm (median 146 cm) and weight ranged from 39-74 kg (median 51kg). Table 1 (see PDF) show demographics and measurements for each patient, TSH and Hb were also measured with height and weight was determined. Serum albumin was measured at baseline where mean albumin levels were 3.23 mg/dl (±0.5). Nutritional status was defined as: well-nourished (>3.5 g/dL), mild malnutrition (3.0-3.5 g/dL) and severe malnutrition (<3.0 g/dL). In addition, L3 SMI and L3 SMA were calculated from CT images. L3 SMI and L3 SMA Details are reported in Table 1 (see PDF). Neuropathy was assessed using nerve conduction velocity studies (NCV) post-cycle 6 and clinical neuropathy was also graded from 0 to 3 based on qualitative reports by the patients (Table 1 - see PDF).

Of the 53 patients with newly diagnosed ovarian carcinoma, 52 of these were epithelial cell carcinoma and 1 was granulosa cell tumor. Twelve of epithelial cell carcinoma was high grade tumors, 7 of the patients had stage 4 diseases and 45 were stage 3 and one patient was stage 1b. The median cumulative paclitaxel dose in neuropathy-positive cases was 1442 mg (±194). In our study by the end of six courses of chemotherapy with paclitaxel and carboplatin, CIPN as documented by NCV was observed in 40% (n=21) of patients while the clinical finding of neuropathy was noted for 43% (n= 23) of patients. Conversely, 32 patients (60%) showed no presence of neuropathy on NCV while the clinical observations showed 57% of patients had no symptoms of neuropathy. However, 30% of patients (n=16) had incongruent findings on NCV and clinical observations. Spearman's rho (0.35) showed poor but significant correlation between the 2 measures of neuropathy (p=0.01) indicating that the clinical grading and objective measurements were often mismatched. Clinical grading of neuropathy identified neuropathy in 9 patients where NCV did not and 7 patients showed presence of neuropathy on NCV in absence of any symptoms. Multivariate analysis of our data showed age as the only independent predictor of neuropathy. In other words, the presence of neuropathies was age dependent such that neuropathies as measured by NCV were present more in older patients. T test showed significant differences between those who showed neuropathies versus those who did not [t test = -4.06, df=51, p <0.001] (Figure 1, 2 - see PDF). No significant correlation was found between L3 SMI and clinical grading of neuropathy (r = 0.10, p = 0.46). L3 SMI also did not differ significantly across nutrition groups (p = 0.75). One way ANOVA showed no significant differences on albumin, Vit B12, L3 SMI, TSH or Hb between those with and without neuropathy as identified by NCV except for the age [(1, 51) = 16.5, p <0.001] and height of the patients [(1, 51) = 4.9, p =0.03]. Further analysis showed that those with neuropathy (n=21) as measured on NCV were noted to be significantly older and taller than those without neuropathy (n =32) (Figure 3 - see PDF). Although the mean differences between the heights was about 4cm and hence needs to be interpreted cautiously. Logistic regression was attempted to determine if any of the considered factors such as age, L3SMI, TSH, Vit B12 level and Hb predict the risk of neuropathy in patients with ovarian cancer. Results showed that the logistic regression model was significant, χ^2^ (4) =31.6, p<0.001. The model explained 78.2% (Nagelkerke R2) of the variance in neuropathy and correctly classified 86.5% of patients. Contrary to the prediction, the measures of nutrition did not contribute to the model.

## Discussion:

In this study 21 patients out of 53 had abnormal NCV. Neuropathy (NCV-documented) was observed in 55.6% of the patients who received six cycles of chemotherapy, namely paclitaxel and carboplatin. In this study, neuropathy was prevalent among 55.6% patients receiving paclitaxel chemotherapy, with a median dose of 1400 ± 194 mg. As per Glare *et al.* (2014) [7]. The prevalence of CIPN ranges from 11% to over 87%. Klein *et al.* (2021) [8] have reported that paclitaxel induced neuropathy may be seen in as many as 97% of all patients treated with paclitaxel. While CIPN's impact on patient's quality of life is well-documented, there is a paucity of knowledge of factors that affect CIPN severity. Age was the only independent predictor of neuropathy in the multivariate analysis done in our study. The presence of neuropathies was age dependent. Neuropathies as measured by NCV were present more in older patients in this study. Our results suggest that host-related factors such as increasing age may play an equally or more significant role. This is also reported by Hurria *et al.* (2016) [9] who reported that older patients treated with chemotherapy have higher rates of severe treatment toxicities. In another study they stated that 60% of older adults experience severe toxicity. Huiqian *et al.* (2025) [10] also found age to be a predominant factor in development of CIPN. Despite previous literature suggesting a link between sarcopenia, malnutrition and chemotherapy-induced peripheral neuropathy (CIPN), our study found no significant association between L3 SMI, albumin, vitamin B12 and neuropathy. Choudhary *et al.* (2022) [11] reported an L3 SMI of 43.93±6.05 in Indian women, with 30.1% of patients having an SMI between 37 and 49, while no significant correlation was found in our cohort. Jin *et al.* (2023) [12] also acknowledged that while sarcopenia is linked to poor outcomes, its role in CIPN remains unclear and the need for standard cutoffs is emphasized. Gwathmey *et al.* (2020) [13] highlighted the need for more understanding of nutrition's role in CIPN. Ottaiano *et al.* (2016) [14] found a positive correlation between obesity and CIPN, with elevated TNF-α and IL-6 contributing to nerve damage. Our study found no correlation between BMI, serum albumin, or B12 levels and neuropathy, which may be due to limitations in sample size. Colvin *et al.* (2021) [15] reported that low albumin levels could be a predictor of CIPN, but our study found no statistical significance. Older age, reduced Hb and TSH levels were associated with an increased likelihood of neuropathy, consistent with findings from Tofthagen *et al.* (2022) [16]. Other factors like chemotherapy doses and duration of infusion, as suggested by Ewertz *et al.* (2015) [17] could contribute to CIPN. NCV identified subclinical neuropathy in patients without overt symptoms, highlighting its role in early detection. Although L3 SMI remains a potential marker for sarcopenia, its lack of correlation with CIPN in this study may be due to the small sample size, varied chemotherapy dosing and multifactorial causes of CIPN. The absence of significant findings does not rule out a true association and future studies with larger sample sizes and adjustments for inflammatory markers are needed.

## Conclusion:

We show no statistically significant association between L3 SMI and neuropathy severity or serum albumin-defined malnutrition. However, the relationships observed warrant further investigation in larger studies with more extensive nutritional and inflammatory assessments. Additionally, NCV was effective in identifying subclinical neuropathy, highlighting its value as an objective tool for early detection, particularly in high-risk or borderline cases.
